# Supplementary material for: Production of a Monoclonal Antibody to the Nucleocapsid Protein of SARS-CoV-2 and Its Application to ELISA-Based Detection Methods with Broad Specificity by Combined Use of Detector Antibodies
Source: Viruses. 2022 Dec 21;15(1):28. doi: 10.3390/v15010028 (PMC9866944; doi:10.3390/v15010028)
Supplement: Supplementary file 1 [file viruses-15-00028-s001.zip › viruses-2076495-supplementary.pdf]

Supplementary Table S1. Raw data of ELISA assay corresponding to Figure 1A

Ascites dilution fold	Replicate 1	Replicate 2	Replicate 3
10000	1.637	1.094	0.941
40000	1.077	0.559	0.325
160000	0.389	0.177	0.13
640000	0.154	0.092	0.078
2560000	0.083	0.066	0.063
10240000	0.065	0.059	0.058
40960000	0.061	0.058	0.056
163840000	0.055	0.052	0.053

Supplementary Table S2. Raw data of ELISA assay corresponding to Figure 1C

Antibody concentration (ng/ml)	Total IgG	IgG1	IgG2a	IgG2b	IgG3
1000	1.6412	0.1363	1.8186	0.1153	0.1337
250	1.5188	0.1330	1.6317	0.1044	0.1100
62.5	1.2474	0.1379	1.3420	0.1028	0.1009
15.625	0.9187	0.1405	0.7826	0.1007	0.0981
3.90625	0.6348	0.1555	0.3741	0.0995	0.0919
0.9765625	0.2981	0.1632	0.1969	0.1009	0.0946
0.244140625	0.2234	0.1420	0.2534	0.0954	0.1015
0	0.1883	0.1437	0.1349	0.1146	0.1482

Supplementary Table S3. Raw data of ELISA assay corresponding to Figure 1D

Antibody concentration (nM)	Normal mIgG				2A7H9			
	Replicate 1	Replicate 2	Avg	Std	Replicate 1	Replicate 2	Avg	Std
1000	0.1093	0.1292	0.1193	0.0141	0.8034	0.6727	0.7381	0.0924
200	0.1034	0.0736	0.0885	0.0211	0.9203	1.0155	0.9679	0.0673
40	0.0568	0.0603	0.0586	0.0025	1.0316	0.9810	1.0063	0.0358
8	0.0574	0.0526	0.0550	0.0034	0.8316	0.9121	0.8719	0.0569
1.6	0.0594	0.0532	0.0563	0.0044	0.4591	0.4533	0.4562	0.0041
0.32	0.0598	0.0524	0.0561	0.0052	0.1920	0.2013	0.1967	0.0066
0.064	0.0563	0.0523	0.0543	0.0028	0.0856	0.0821	0.0839	0.0025
0.0128	0.0546	0.0543	0.0545	0.0002	0.0621	0.0621	0.0621	0.0000
0.00256	0.0544	0.0543	0.0544	0.0001	0.0546	0.0564	0.0555	0.0013
0.000512	0.0573	0.0584	0.0579	0.0008	0.0592	0.0542	0.0567	0.0035
0.0001024	0.0584	0.0564	0.0574	0.0014	0.0651	0.0600	0.0626	0.0036
0.00002048	0.0587	0.0550	0.0569	0.0026	0.0594	0.0586	0.0590	0.0006

Supplementary Table S4. Raw data of ELISA assay corresponding to Figure 2A

Antigen concentration (ng/well)	Normal mIgG					1G10C4					2A7H9				
	Replicate 1	Replicate 2	Replicate 3	Avg	Std	Replicate 1	Replicate 2	Replicate 3	Avg	Std	Replicate 1	Replicate 2	Replicate 3	Avg	Std
10	0.0835	0.0710	0.0615	0.0720	0.0110	2.1797	1.3282	1.0484	1.5188	0.5892	0.8395	0.9570	1.3398	1.0454	0.2616
3.333	0.0711	0.0572	0.0568	0.0617	0.0081	1.2119	0.9239	0.7807	0.9722	0.2196	0.4379	0.4894	0.6281	0.5185	0.0984
1.111	0.0605	0.0574	0.0535	0.0571	0.0035	0.4289	0.3560	0.2861	0.3570	0.0714	0.1941	0.2221	0.2971	0.2378	0.0533
0.370	0.0595	0.0550	0.0546	0.0564	0.0027	0.2266	0.1878	0.1585	0.1910	0.0342	0.1099	0.1022	0.1219	0.1113	0.0099
0.123	0.0590	0.0587	0.0535	0.0571	0.0031	0.1184	0.1023	0.0922	0.1043	0.0132	0.0692	0.0657	0.0759	0.0703	0.0052
0.041	0.0609	0.0538	0.0520	0.0556	0.0047	0.0793	0.0707	0.0639	0.0713	0.0077	0.0552	0.0573	0.0600	0.0575	0.0024
0.014	0.0607	0.0542	0.0528	0.0559	0.0042	0.0671	0.0589	0.0554	0.0605	0.0060	0.0517	0.0557	0.0616	0.0563	0.0050
0.005	0.0657	0.0574	0.0532	0.0588	0.0064	0.0605	0.0573	0.0533	0.0570	0.0036	0.0556	0.0548	0.0716	0.0607	0.0095

Supplementary Table S5. Raw data of ELISA assay corresponding to Figure 2B

Antibody concentration (ng/well)	Normal mIgG					1G10C4					2A7H9				
	Replicate 1	Replicate 2	Replicate 3	Avg	Std	Replicate 1	Replicate 2	Replicate 3	Avg	Std	Replicate 1	Replicate 2	Replicate 3	Avg	Std
10	0.0577	0.0632	0.0649	0.0619	0.0038	1.8957	1.8521	2.1837	1.9772	0.1802	1.7057	1.6265	1.7134	1.6819	0.0481
3.333	0.0542	0.0543	0.0566	0.0550	0.0014	1.4601	1.4824	1.5982	1.5136	0.0741	1.4013	1.4798	1.5851	1.4887	0.0922
1.111	0.0517	0.0522	0.0559	0.0533	0.0023	0.6679	0.7997	0.8762	0.7813	0.1054	0.8139	0.8628	1.0868	0.9212	0.1455
0.370	0.0504	0.0518	0.0576	0.0533	0.0038	0.2466	0.3023	0.3166	0.2885	0.0370	0.3506	0.3898	0.4694	0.4033	0.0605
0.123	0.0551	0.0503	0.0499	0.0518	0.0029	0.0933	0.1323	0.1366	0.1207	0.0239	0.1266	0.1558	0.1806	0.1543	0.0270
0.041	0.0499	0.0517	0.0504	0.0507	0.0009	0.0682	0.0792	0.0757	0.0744	0.0056	0.0717	0.0719	0.0988	0.0808	0.0156
0.014	0.0515	0.0552	0.0516	0.0528	0.0021	0.0653	0.0604	0.0604	0.0620	0.0028	0.0596	0.0594	0.0628	0.0606	0.0019
0.005	0.0519	0.0516	0.0524	0.0520	0.0004	0.0558	0.0572	0.0562	0.0564	0.0007	0.0546	0.0551	0.0686	0.0594	0.0079

Supplementary Table S6. Raw data of ELISA assay corresponding to Figure 2C

Antibody concentration (ng/well)	Mouse IgG2a				Normal mIgG				Normal hIgG			
	Replicate 1	Replicate 2	Avg	Std	Replicate 1	Replicate 2	Avg	Std	Replicate 1	Replicate 2	Avg	Std
10	0.0535	0.0565	0.0550	0.0021	0.0575	0.0535	0.0555	0.0028	0.0617	0.0604	0.0611	0.0009
3.333	0.0555	0.0518	0.0537	0.0026	0.0540	0.0536	0.0538	0.0003	0.0605	0.0579	0.0592	0.0018
1.111	0.0529	0.0559	0.0544	0.0021	0.0542	0.0533	0.0538	0.0006	0.0549	0.0545	0.0547	0.0003
0.370	0.0547	0.0535	0.0541	0.0008	0.0527	0.0540	0.0534	0.0009	0.0578	0.0519	0.0549	0.0042
0.123	0.0539	0.0545	0.0542	0.0004	0.0533	0.0574	0.0554	0.0029	0.0540	0.0546	0.0543	0.0004
0.041	0.0548	0.0733	0.0641	0.0131	0.0530	0.0529	0.0530	0.0001	0.0554	0.0536	0.0545	0.0013
0.014	0.0625	0.0545	0.0585	0.0057	0.0549	0.0656	0.0603	0.0076	0.0668	0.0523	0.0596	0.0103
0.005	0.0540	0.0520	0.0530	0.0014	0.0521	0.0526	0.0524	0.0004	0.0553	0.0535	0.0544	0.0013

Supplementary Table S7. Raw data of ELISA assay corresponding to Figure 3A

Antigen concentration (ng/well)	1G10C4 mAb-bio					2A7H9 mAb-bio					Mouse IgG2a-bio				
	Replicate 1	Replicate 2	Replicate 3	Avg	Std	Replicate 1	Replicate 2	Replicate 3	Avg	Std	Replicate 1	Replicate 2	Replicate 3	Avg	Std
10	1.4235	1.2844	1.3516	1.3532	0.0696	1.6671	1.6691	1.4368	1.5910	0.1335	0.0720	0.0748	0.0739	0.0736	0.0014
3.333	0.7574	0.7191	0.7852	0.7539	0.0332	1.1922	1.2055	0.9892	1.1290	0.1212	0.0993	0.0736	0.0733	0.0821	0.0149
1.111	0.3757	0.3478	0.3775	0.3670	0.0167	0.6886	0.7760	0.8418	0.7688	0.0769	0.0699	0.0786	0.0694	0.0726	0.0052
0.370	0.1645	0.1738	0.1895	0.1759	0.0126	0.4951	0.4890	0.4610	0.4817	0.0182	0.0752	0.0703	0.0752	0.0736	0.0028
0.123	0.0917	0.0982	0.1037	0.0979	0.0060	0.2238	0.2483	0.2592	0.2438	0.0181	0.0810	0.0708	0.0741	0.0753	0.0052
0.041	0.0671	0.0683	0.0719	0.0691	0.0025	0.1164	0.1231	0.1157	0.1184	0.0041	0.0869	0.0772	0.0797	0.0813	0.0050
0.014	0.0602	0.0663	0.0650	0.0638	0.0032	0.0773	0.0770	0.0787	0.0777	0.0009	0.0936	0.0936	0.0816	0.0896	0.0069
0.005	0.0592	0.0618	0.0622	0.0611	0.0016	0.0653	0.0659	0.0641	0.0651	0.0009	0.1024	0.0890	0.1020	0.0978	0.0076

Supplementary Table S8. Raw data of ELISA assay corresponding to Figure 3B

Antigen concentration (ng/well)	1G10C4 mAb-bio					2A7H9 mAb-bio					Mouse IgG2a-bio				
	Replicate 1	Replicate 2	Replicate 3	Avg	Std	Replicate 1	Replicate 2	Replicate 3	Avg	Std	Replicate 1	Replicate 2	Replicate 3	Avg	Std
10	1.6355	1.6874	1.6001	1.6410	0.0439	1.2079	1.2980	1.3145	1.2735	0.0574	0.0668	0.0722	0.0789	0.0726	0.0061
3.333	1.4643	1.2064	1.2887	1.3198	0.1317	0.7639	0.7564	0.6701	0.7301	0.0521	0.0706	0.0703	0.0642	0.0684	0.0036
1.111	0.9344	0.8833	0.9219	0.9132	0.0266	0.2519	0.3782	0.3626	0.3309	0.0689	0.0589	0.0651	0.0616	0.0619	0.0031
0.370	0.6129	0.6326	0.5406	0.5954	0.0484	0.1334	0.1306	0.1563	0.1401	0.0141	0.0607	0.0647	0.0574	0.0609	0.0037
0.123	0.2484	0.2611	0.2171	0.2422	0.0226	0.0777	0.0826	0.0781	0.0795	0.0027	0.0656	0.0533	0.0628	0.0606	0.0064
0.041	0.1278	0.1212	0.1270	0.1253	0.0036	0.0679	0.0716	0.0642	0.0679	0.0037	0.0654	0.0615	0.0623	0.0631	0.0021
0.014	0.0751	0.0801	0.0808	0.0787	0.0031	0.0649	0.0640	0.0666	0.0652	0.0013	0.0626	0.0601	0.0673	0.0633	0.0037
0.005	0.0634	0.0678	0.0639	0.0650	0.0024	0.0605	0.0602	0.0627	0.0611	0.0014	0.0645	0.0605	0.0582	0.0611	0.0032

Supplementary Table S9. Raw data of ELISA assay corresponding to Figure 3C

Antigen concentration (ng/well)	1G10C4 mAb-bio					2A7H9 mAb-bio					Mouse IgG2a-bio				
	Replicate 1	Replicate 2	Replicate 3	Avg	Std	Replicate 1	Replicate 2	Replicate 3	Avg	Std	Replicate 1	Replicate 2	Replicate 3	Avg	Std
10	0.0573	0.0653	0.0513	0.0580	0.0070	0.0573	0.0527	0.0595	0.0565	0.0035	0.0584	0.0552	0.0563	0.0566	0.0016
3.333	0.0529	0.0544	0.0515	0.0529	0.0015	0.0505	0.0495	0.0511	0.0504	0.0008	0.0527	0.0521	0.0571	0.0540	0.0027
1.111	0.0500	0.0534	0.0529	0.0521	0.0018	0.0520	0.0508	0.0498	0.0509	0.0011	0.0511	0.0530	0.0527	0.0523	0.0010
0.370	0.0504	0.0506	0.0501	0.0504	0.0003	0.0509	0.0524	0.0514	0.0516	0.0008	0.0537	0.0540	0.0567	0.0548	0.0017
0.123	0.0517	0.0530	0.0501	0.0516	0.0015	0.0553	0.0503	0.0511	0.0522	0.0027	0.0533	0.0533	0.0552	0.0539	0.0011
0.041	0.0564	0.0479	0.0500	0.0514	0.0044	0.0524	0.0493	0.0485	0.0501	0.0021	0.0501	0.0516	0.0548	0.0522	0.0024
0.014	0.0589	0.0521	0.0520	0.0543	0.0040	0.0513	0.0522	0.0512	0.0516	0.0006	0.0556	0.0543	0.0559	0.0553	0.0009
0.005	0.0504	0.0504	0.0512	0.0507	0.0005	0.0538	0.0507	0.0520	0.0522	0.0016	0.0555	0.0531	0.0586	0.0557	0.0028

Supplementary Table S10. Raw data of ELISA assay corresponding to Figure 3D

Antigen concentration (ng/well)	1G10C4 mAb-bio					2A7H9 mAb-bio					Mouse IgG2a-bio				
	Replicate 1	Replicate 2	Replicate 3	Avg	Std	Replicate 1	Replicate 2	Replicate 3	Avg	Std	Replicate 1	Replicate 2	Replicate 3	Avg	Std
10	0.0496	0.0521	0.0498	0.0505	0.0014	0.0543	0.0553	0.0545	0.0547	0.0005	0.0549	0.0547	0.0552	0.0549	0.0003
3.333	0.0500	0.0510	0.0529	0.0513	0.0015	0.0539	0.0510	0.0539	0.0529	0.0017	0.0568	0.0547	0.0615	0.0577	0.0035
1.111	0.0498	0.0521	0.0512	0.0510	0.0012	0.0510	0.0555	0.0518	0.0528	0.0024	0.0552	0.0538	0.0543	0.0544	0.0007
0.370	0.0497	0.0507	0.0518	0.0507	0.0011	0.0513	0.0524	0.0529	0.0522	0.0008	0.0547	0.0758	0.0570	0.0625	0.0116
0.123	0.0528	0.0499	0.0550	0.0526	0.0026	0.0505	0.0512	0.0517	0.0511	0.0006	0.0543	0.0570	0.0553	0.0555	0.0014
0.041	0.0507	0.0496	0.0532	0.0512	0.0018	0.0537	0.0496	0.0490	0.0508	0.0026	0.0542	0.0562	0.0547	0.0550	0.0010
0.014	0.0498	0.0498	0.0549	0.0515	0.0029	0.0518	0.0573	0.0508	0.0533	0.0035	0.0576	0.0832	0.0547	0.0652	0.0157
0.005	0.0497	0.0528	0.0510	0.0512	0.0016	0.0506	0.0508	0.0503	0.0506	0.0003	0.0565	0.0573	0.0550	0.0563	0.0012

Supplementary Table S11. Raw data of ELISA assay corresponding to Figure 4A

Virus (pfu/well)	Mock				Omicron			
	Replicate 1	Replicate 2	Avg	Std	Replicate 1	Replicate 2	Avg	Std
20	0.0630	0.0630	0.0630	0.0000	0.1548	0.2192	0.1870	0.0455
6.667	0.0605	0.0604	0.0605	0.0001	0.1385	0.1515	0.1450	0.0092
2.222	0.1372	0.0618	0.0995	0.0533	0.1056	0.1047	0.1052	0.0006
0.741	0.0604	0.0637	0.0621	0.0023	0.0837	0.0857	0.0847	0.0014
0.247	0.0608	0.0634	0.0621	0.0018	0.0751	0.0731	0.0741	0.0014
0.082	0.0622	0.0637	0.0630	0.0011	0.0751	0.0753	0.0752	0.0001
0.027	0.0605	0.0619	0.0612	0.0010	0.0694	0.0751	0.0723	0.0040
0.009	0.0590	0.0604	0.0597	0.0010	0.0793	0.0795	0.0794	0.0001

Supplementary Table S12. Raw data of ELISA assay corresponding to Figure 5A

Virus (pfu/well)	Mock				S clade			
	Replicate 1	Replicate 2	Avg	Std	Replicate 1	Replicate 2	Avg	Std
20	0.0475	0.0514	0.0495	0.0028	0.2065	0.2328	0.2197	0.0186
6.667	0.0496	0.0482	0.0489	0.0010	0.1951	0.1739	0.1845	0.0150
2.222	0.0501	0.0503	0.0502	0.0001	0.1225	0.1311	0.1268	0.0061
0.741	0.0494	0.0503	0.0499	0.0006	0.0768	0.0830	0.0799	0.0044
0.247	0.0575	0.0487	0.0531	0.0062	0.0693	0.0592	0.0643	0.0071
0.082	0.0480	0.0495	0.0488	0.0011	0.0528	0.0526	0.0527	0.0001
0.027	0.0517	0.0511	0.0514	0.0004	0.0511	0.0553	0.0532	0.0030
0.009	0.0501	0.0492	0.0497	0.0006	0.0495	0.0530	0.0513	0.0025
Virus (pfu/well)	Delta				Iota			
	Replicate 1	Replicate 2	Avg	Std	Replicate 1	Replicate 2	Avg	Std
20	0.2550	0.2988	0.2769	0.0310	0.6466	0.8726	0.7596	0.1598
6.667	0.1573	0.1841	0.1707	0.0190	0.3968	0.4137	0.4053	0.0120
2.222	0.0896	0.1163	0.1030	0.0189	0.1752	0.1752	0.1752	0.0000
0.741	0.0643	0.0705	0.0674	0.0044	0.0810	0.0912	0.0861	0.0072
0.247	0.0546	0.0592	0.0569	0.0033	0.2235	0.1715	0.1975	0.0368
0.082	0.0582	0.0566	0.0574	0.0011	0.0846	0.0557	0.0702	0.0204
0.027	0.0505	0.0535	0.0520	0.0021	0.0520	0.0577	0.0549	0.0040
0.009	0.0505	0.0513	0.0509	0.0006	0.0533	0.0518	0.0526	0.0011
Virus (pfu/well)	Mu				Omicron			
	Replicate 1	Replicate 2	Avg	Std	Replicate 1	Replicate 2	Avg	Std
20	0.9793	1.0732	1.0263	0.0664	1.0936	1.0117	1.0527	0.0579
6.667	0.8008	0.8113	0.8061	0.0074	0.6136	0.6843	0.6490	0.0500
2.222	0.4381	0.4107	0.4244	0.0194	0.2620	0.3022	0.2821	0.0284
0.741	0.1315	0.1597	0.1456	0.0199	0.1169	0.1209	0.1189	0.0028
0.247	0.0874	0.0916	0.0895	0.0030	0.0746	0.0757	0.0752	0.0008
0.082	0.0654	0.0642	0.0648	0.0008	0.0630	0.0609	0.0620	0.0015
0.027	0.0548	0.0571	0.0560	0.0016	0.0590	0.0580	0.0585	0.0007
0.009	0.0539	0.0561	0.0550	0.0016	0.0631	0.0699	0.0665	0.0048

Supplementary Table S13. Raw data of ELISA assay corresponding to Figure 5B

Virus (pfu/well)	Mock				S clade			
	Replicate 1	Replicate 2	Avg	Std	Replicate 1	Replicate 2	Avg	Std
20	0.0580	0.0575	0.0578	0.0004	1.2039	0.8240	1.0140	0.2686
6.667	0.0596	0.0588	0.0592	0.0006	0.8271	0.8060	0.8166	0.0149
2.222	0.0556	0.0597	0.0577	0.0029	0.4163	0.4788	0.4476	0.0442
0.741	0.0653	0.0582	0.0618	0.0050	0.2135	0.2534	0.2335	0.0282
0.247	0.0664	0.0600	0.0632	0.0045	0.1185	0.1203	0.1194	0.0013
0.082	0.0582	0.0572	0.0577	0.0007	0.0793	0.0829	0.0811	0.0025
0.027	0.0599	0.0589	0.0594	0.0007	0.0617	0.0641	0.0629	0.0017
0.009	0.0595	0.0591	0.0593	0.0003	0.0583	0.0578	0.0581	0.0004

Virus (pfu/well)	Delta				Iota			
	Replicate 1	Replicate 2	Avg	Std	Replicate 1	Replicate 2	Avg	Std
20	0.6888	0.8176	0.7532	0.0911	0.6095	0.7697	0.6896	0.1133
6.667	0.5176	0.5362	0.5269	0.0132	0.3797	0.2645	0.3221	0.0815
2.222	0.1859	0.2400	0.2130	0.0383	0.1773	0.1501	0.1637	0.0192
0.741	0.1122	0.1153	0.1138	0.0022	0.1020	0.0856	0.0938	0.0116
0.247	0.0781	0.0737	0.0759	0.0031	0.0667	0.0645	0.0656	0.0016
0.082	0.0589	0.0584	0.0587	0.0004	0.0603	0.0569	0.0586	0.0024
0.027	0.0536	0.0551	0.0544	0.0011	0.0542	0.0534	0.0538	0.0006
0.009	0.0583	0.0557	0.0570	0.0018	0.0578	0.0519	0.0549	0.0042

Virus (pfu/well)	Mu				Omicron			
	Replicate 1	Replicate 2	Avg	Std	Replicate 1	Replicate 2	Avg	Std
20	0.6787	0.4769	0.5778	0.1427	0.3889	0.3946	0.3918	0.0040
6.667	0.5087	0.4035	0.4561	0.0744	0.2333	0.2175	0.2254	0.0112
2.222	0.2556	0.1597	0.2077	0.0678	0.1209	0.1084	0.1147	0.0088
0.741	0.1378	0.1059	0.1219	0.0226	0.0717	0.0721	0.0719	0.0003
0.247	0.0839	0.0699	0.0769	0.0099	0.0624	0.0593	0.0609	0.0022
0.082	0.0626	0.0621	0.0624	0.0004	0.0551	0.0549	0.0550	0.0001
0.027	0.0616	0.0551	0.0584	0.0046	0.0531	0.0529	0.0530	0.0001
0.009	0.0527	0.0565	0.0546	0.0027	0.0505	0.0564	0.0535	0.0042

Supplementary Table S14. Raw data of ELISA assay corresponding to Figure 5C

Virus (pfu/well)	Mock				S clade			
	Replicate 1	Replicate 2	Avg	Std	Replicate 1	Replicate 2	Avg	Std
20	0.0710	0.0715	0.0713	0.0004	2.0755	1.8065	1.9410	0.1902
6.667	0.0718	0.0726	0.0722	0.0006	1.4764	1.2851	1.3808	0.1353
2.222	0.0690	0.0764	0.0727	0.0052	0.7373	0.6942	0.7158	0.0305
0.741	0.0767	0.0743	0.0755	0.0017	0.2174	0.2107	0.2141	0.0047
0.247	0.0793	0.0779	0.0786	0.0010	0.1206	0.1202	0.1204	0.0003
0.082	0.0664	0.0743	0.0704	0.0056	0.0856	0.0830	0.0843	0.0018
0.027	0.0732	0.0719	0.0726	0.0009	0.0738	0.0775	0.0757	0.0026
0.009	0.0696	0.0757	0.0727	0.0043	0.0746	0.0789	0.0768	0.0030

Virus (pfu/well)	Delta				Iota			
	Replicate 1	Replicate 2	Avg	Std	Replicate 1	Replicate 2	Avg	Std
20	0.9669	0.7250	0.8460	0.1710	0.6525	0.6859	0.6692	0.0236
6.667	0.5998	0.5137	0.5568	0.0609	0.4175	0.4350	0.4263	0.0124
2.222	0.2945	0.2623	0.2784	0.0228	0.2086	0.2340	0.2213	0.0180
0.741	0.1340	0.1301	0.1321	0.0028	0.1153	0.1340	0.1247	0.0132
0.247	0.0898	0.0845	0.0872	0.0037	0.0835	0.0947	0.0891	0.0079
0.082	0.0787	0.0837	0.0812	0.0035	0.0736	0.0767	0.0752	0.0022
0.027	0.0741	0.0733	0.0737	0.0006	0.0722	0.0742	0.0732	0.0014
0.009	0.0783	0.0738	0.0761	0.0032	0.0683	0.0717	0.0700	0.0024

Virus (pfu/well)	Mu				Omicron			
	Replicate 1	Replicate 2	Avg	Std	Replicate 1	Replicate 2	Avg	Std
20	1.0094	1.2787	1.1441	0.1904	0.0817	0.0835	0.0826	0.0013
6.667	0.7968	1.0203	0.9086	0.1580	0.0751	0.0770	0.0761	0.0013
2.222	0.4546	0.6230	0.5388	0.1191	0.0744	0.0733	0.0739	0.0008
0.741	0.1949	0.2636	0.2293	0.0486	0.0743	0.0717	0.0730	0.0018
0.247	0.1161	0.1401	0.1281	0.0170	0.0725	0.0693	0.0709	0.0023
0.082	0.0862	0.0984	0.0923	0.0086	0.0767	0.0704	0.0736	0.0045
0.027	0.0793	0.0861	0.0827	0.0048	0.0776	0.0779	0.0778	0.0002
0.009	0.0756	0.0865	0.0811	0.0077	0.0846	0.0788	0.0817	0.0041
